# Abiotic Stresses: Insight into Gene Regulation and Protein Expression in Photosynthetic Pathways of Plants

**DOI:** 10.3390/ijms160920392

**Published:** 2015-08-28

**Authors:** Mohammad-Zaman Nouri, Ali Moumeni, Setsuko Komatsu

**Affiliations:** 1 Rice Research Institute of Iran, Mazandaran Branch, Agricultural Research, Education and Extension Organization (AREEO), Amol 46191-91951, Iran; E-Mail: amoumeni@areo.ir; 2 National Institute of Crop Science, National Agriculture and Food Research Organization, Tsukuba 305-8518, Japan

**Keywords:** abiotic stress, photosynthesis, gene regulation, protein expression

## Abstract

Global warming and climate change intensified the occurrence and severity of abiotic stresses that seriously affect the growth and development of plants, especially, plant photosynthesis. The direct impact of abiotic stress on the activity of photosynthesis is disruption of all photosynthesis components such as photosystem I and II, electron transport, carbon fixation, ATP generating system and stomatal conductance. The photosynthetic system of plants reacts to the stress differently, according to the plant type, photosynthetic systems (C_3_ or C_4_), type of the stress, time and duration of the occurrence and several other factors. The plant responds to the stresses by a coordinate chloroplast and nuclear gene expression. Chloroplast, thylakoid membrane, and nucleus are the main targets of regulated proteins and metabolites associated with photosynthetic pathways. Rapid responses of plant cell metabolism and adaptation to photosynthetic machinery are key factors for survival of plants in a fluctuating environment. This review gives a comprehensive view of photosynthesis-related alterations at the gene and protein levels for plant adaptation or reaction in response to abiotic stress.

## 1. Introduction

Abiotic stresses are major constraints to all living organisms with more challenges to the plants, as they cannot move as other organisms [1]. Several reports indicated that the structure of living macromolecules such as lipids, proteins, and nucleic acids are disposed to damage and/or degradation under severe abiotic stress conditions [2]. From an agricultural context, abiotic stresses are ultimately defined in terms of their effects on crop yield as final economic output [3]. Despite that plant growth is controlled by a variety of physiological, biochemical, and molecular processes, photosynthesis is a key mechanism, which provides to a large extent energy as well as organic molecules for plant growth and development [4]. Generally in the plant kingdom and specifically in higher plants, leaves serve as a highly specialized part that is basically appointed in the photosynthetic process [5]. Photosynthesis represents one of the most important photo-chemical reactions in plants, since energy from sunlight is trapped and converted into biological energy. Hence, improving the efficiency of photosynthesis could have a huge beneficial impact [6]. Photosynthesis is a consequence of a multi-step and complicated process that involves several biological pathways. The Pathways are photosynthetic electron transport system (PETs), in which the light energy is altered into ATP and NADPH; The Calvin–Benson cycle that is also known as a photosynthetic carbon fixation cycle in which CO_2_ is fixed into carbohydrates, as well as assimilation, transport, and utilization of photoassimilates as the organic products of photosynthesis [7,8,9].

The two important steps, PETs and the Calvin–Benson cycle, are under the control of many genes/gene products encoded from chloroplast as well as nuclear genomes. While the products of genes involved in photosynthesis have obvious functions, they operate together within the framework of an extensively coordinated photosynthetic network of genes, regulatory components, signaling factors, and metabolic processes. The expression of genes in both cellular organelles is highly variable and affected by a diverse range of environmental factors [10]. Many environmental stresses such as drought, salinity, flooding, light, unfavorable temperatures, and its rapid fluctuations adversely affect the process of photosynthetic carbon metabolism in plants. It may alter the ultrastructure of the organelles, change the concentration of various pigments and metabolites as well as stomatal regulation [3,11]. Several reports indicate that photosynthesis cascades are highly correlated with the accumulation of some important proteins such as ribulose-1,5-bisphosphate carboxylase/oxygenase (RuBisCO) and other photosynthesis-related proteins [12,13].

To get insight into the photosynthetic gene expression and regulation under abiotic stresses, OMICS technologies such as genomics, transcriptomics, proteomics, and metabolomics can provide detailed information which can be later applied to improve plant yield potentials. In response to various abiotic stresses plants continuously need to adjust their transcriptome profile [1]. In recent decades, transcriptomic and proteomic approaches have emerged as powerful tools to analyze genome expression at the transcription and translational levels, respectively [14]. These high-throughput technologies have been extensively accepted to study the expression of certain genes and proteins in response to different abiotic stresses [15]. Proteomics, as one of the cutting edge molecular techniques, efficiently deals with the functional molecular studies. Recently, improvement of techniques for isolation and purification of cell organelles and compartments gave new insights into organelle proteomics [16].

Photosynthesis in plants is under the control of a complex network of proteins. Four major multisubunit protein complexes, photosystem (PS) I, PSII, the ATP synthase complex and cytochrome *b_6_*/*f* complex are involved in the process [17]. These proteins are greatly affected under abiotic stress conditions. This review paper provides an overview of the effect of abiotic stresses on gene regulation and protein expression involved in photosynthesis in plants with emphasis on the data reported through transcriptome and proteome technologies. It describes molecular mechanisms that determine how these different classes of genes and proteins are regulated in response to abiotic stress conditions.

## 2. Photosynthesis in C_3_ and C_4_ Plants in Response to Elevated CO_2_ Concentration

### 2.1. C_3_ Plants

Plants with the metabolic pathways of C_3_ for carbon fixation are distributed worldwide. They represent over 95% of the earth’s plant species, especially in cold and wet climates, usually with low light intensity. In C_3_ plants, the photosynthetic Carbon Reduction or Calvin–Benson cycle for CO_2_ fixation produces a three-carbon compound, phosphoglycerate. Therefore, plants utilizing this pathway are often named as C_3_ species [18]. According to a systems biology analysis, the photosynthetic metabolism of C_3_ plants has a highly cooperative regulation in changing environments [19]. Effects of environmental changes and abiotic stresses on photosynthesis system of many C_3_ plants, from stomatal conductance to carbon assimilation and from gene regulation to protein expression are well documented [3,20]. Various components are involved in the mechanism of photosynthesis in response to environmental stresses, including photosynthetic pigments and photosystems, the electron transport system, and CO_2_ reduction pathways.

Changes in CO_2_ level of atmosphere is an environmental factor with the most direct and instant effect on photosynthesis. Global atmospheric CO_2_ concentration of the earth is 380 μL/L which is 40% more than pre-industrial times. Values are predicted to reach between 530 and 970 μL/L by the end of this century [21]. In theory, elevated CO_2_ will directly affect the balance between photosynthetic carbon fixation and photorespiration. However, plant response to high CO_2_ is under the influence of several factors, including plant carbon fixation pathways. Foyer *et al.* [9] reviewed the literature related to the C_3_ and C_4_ plant responses to elevated CO_2_ concentration compared with those grown with ambient CO_2_ [9]. Exposing C_3_ leaves to high CO_2_, immediately increases net photosynthesis because of decreased photorespiration [22,23] and enhances the expression of genes associated with cyclic electron flow pathways. However, long-term elevated CO_2_ often decreases photosynthetic capacity, RuBisCO activity and CO_2_ fixation [9].

### 2.2. C_4_ Plants

C_4_ plants are named for the four-carbon organic acids produced in the first product of carbon fixation. C_4_ plants have an improved photosynthetic efficiency with minimized water loss in hot and dry environments. Generally, these kind of species are native to the tropics and warmer climates with high light intensity exhibiting a higher photosynthetic and growth rate due to gains in the water, carbon and nitrogen efficiency uses [24]. Maize (*Zea mays*), sugar cane (*Saccharum officinarum*) and sorghum (*Sorghum bicolor*) are among the most productive crops with C_4_ photosynthesis pathway [25]. Although, C_3_ and C_4_ plants are alike in the basic photosynthetic pathways such as Calvin–Benson cycle and electron transport chain components, significant differences exist in their response to environmental changes.

Response of C_4_ plants to elevated CO_2_ concentration is not similar to those in C_3_ plants. C_4_ species have greater rates of CO_2_ assimilation for a given leaf nitrogen [25]. The association of photosynthesis rate and intercellular CO_2_ concentration was compared in soybean (C_3_) and corn (C_4_). The CO_2_ concentration of 384 µmol/mol as the ambient level of 2009 was compared with 700 µmol/mol for the predicted concentration at the end of this century. According to the results, while photosynthesis was stimulated by 39% in soybean, there was no change in the photosynthesis rate of corn under elevated CO_2_ concentration [26]. Ghannoum [27] reviewed the C_4_ photosynthesis response to water stress and in interaction with CO_2_ concentration and emphasized that elevated CO_2_ concentration alleviates the deleterious effect of drought on plant productivity. It is well known that abiotic stresses such as drought, reduces stomatal conductance, CO_2_ assimilation rate, and intercellular CO_2_ [27,28]. Therefore, saturating CO_2_ concentration keeps the photosynthetic capacity unchanged.

## 3. Impact of Abiotic Stress on the Photosynthetic System of Plants

Abiotic stresses such as drought, cold, salinity, high temperature and so on can adversely affect growth and productivity of plants. Hence, an overview of the effect of these stresses is presented on the profile of gene expression/protein abundance of photosynthesis related pathways and their regulation networks in plants.

### 3.1. Drought

Drought has been recognized as a primary constraint in limiting the growth and development of plants. It usually causes loss of water content, reduced leaf water potential, stomatal conductance, and transpiration rate [18]. Stomatal closure is the earliest response to drought causing a decrease in mesophyll CO_2_ diffusion and reduction in the photosynthesis rate [29]. In crop plants, decrease in carbon gain through photosynthesis is the major reason for loss of yield under drought. Drought stress has been shown to inhibit photosynthesis in plants within a few days of limiting water supply, thereby causing a significant reduction in CO_2_ assimilation rate [30]. Decrease in photosynthesis under stress, reduces utilization of absorbed light energy in chloroplasts and the excess light energy could lead to photoinhibition. Photoinhibition, reduces quantum yield of PSII and induces photorespiration and H_2_O_2_ production [31,32]. In this situation, plants can increase utilization of absorbed light energy by improving CO_2_ fixation to minimize photoinhibition [33]. Prolonged and severe drought stress will result in interruption of the energy production process and metabolism and ultimately cell death.

Gene expression profiles of model plants from mocots and dicots includingrice [34,35,36,37,38], and Arabidopsis [39,40] in response to drought stress were extensively studied using heterogenous genotypes [41,42,43,44,45,46] or near isogenic lines [36] at both vegetative and reproductive stages through transcriptome analysis with single or multiple stress treatments. Results from these experiments indicated that most genes involved in photosynthesis are down-regulated in response to drought stress treatments. Chlorophyll a/b-binding protein CP24, PSI reaction center subunit V, protochlorophyllide reductase A, peptidyl-prolyl cis-trans isomerase, and others functioning in the photosynthetic pathways are examples of down-regulated genes in rice leaf tissues when subjected to drought stress [37]. Gene expression profiling of physic nut (*Jatropha curcas* L.) seedlings exposed to drought indicated that the expression of genes involved in PSI, PSII and Calvin cycle components such as light-harvesting complex proteins, and genes encoding key enzymes in the Calvin cycle, RuBisCO small subunit, phosphoglycerate kinase and phosphoribulokinase were significantly down-regulated. However, several genes encoding glycolysis and the TCA cycle, including 6-phosphofructokinase, aconitate hydratase, and dihydrolipoamide succinyltransferase were up-regulated [47]. The evidence from these reports showed that inhibition of photosynthesis is the major consequence of drought reaction in rice leaf.

Gene expression analyses in tolerant genotypes of C_3_ and C_4_ plants were already reported. Genes encoding components of PSI, PSII as well as several genes related to the Calvin–Benson cycle, such as triosephosphate isomerase, fructose-1,6-bisphosphatase, RuBisCO small subunit and RuBisCO activase were repressed in tolerantrice genotypes, such as a C_3_ plant [36,41] and in tolerant maize genotypes, as a C_4_ plant [48] in response to drought stress. There were several common genes related to photosynthesis, which were down-regulated in tolerant and susceptible genotypes in response to drought stress. Therefore, it can be assumed that plants avoid photo-oxidation of the photosynthetic machinery and the creation of free radicals that are destructive for the cell.

In the meantime, activation of several key genes at different cycles of the photosynthetic pathways were reported in rice [36], maize [48] and Arabiodopsis [49]. Some of the activated gene transcripts were PSII (P680 chlorophyll a, LOC_Os07g01480) from photosystem and electron transport, phosphoglycolate phosphatases (LOC_Os03g24070) from photorespiration, and RuBisCO (LOC_Os03g09090) together with eight more gene transcripts from Calvin–Benson cycle which was reported in two near-isogenic lines (NILs) of rice against water-deficit treatments (Table 1). Reduction in photosynthetic activity in response to drought stress is due to a decline in stomatal conductance as well as RuBisCO activities resulting in lower carbon fixation followed by the over-reduction of components of the electron transport system and production of reactive oxygen species.

**Table 1:** Significant differentially expressed genes involved in photosynthesis pathway in rice genotypes including two pairs of near-isogenic lines (NILs) and their susceptible parent (IR64) in response to severe drought stress [42].

Photosynthesis Cycle	Locus_ID	Reaction ID	Gene Expression, log_2_ratio *				
			IR64 †	NIL10	NIL13	NIL11	NIL18
Calvin cycle	LOC_Os03g56869	ribose-5-phosphate isomerase	−3.465	−3.211	−3.139	−3.229	−2.624
	LOC_Os07g08030	ribose-5-phosphate isomerase	−3.844	−3.463	−3.263	−3.015	−2.710
	LOC_Os04g50880	uridine kinase	−3.140	−3.140	−2.952	−2.185	−2.102
	LOC_Os02g47020	uridine kinase	−2.574	−2.495	−3.062	−2.185	−1.861
	LOC_Os03g07300	orotidine-5ʹ-phosphate decarboxylase	−2.570	−2.449	−2.164	−1.826	−1.507
	LOC_Os06g04270	Transketolase	−1.844	−1.333	−1.390	−1.394	−1.293
	LOC_Os04g19740	Transketolase	3.525	3.068	2.905	3.630	3.341
	LOC_Os06g04270	Transketolase	−1.844	−1.333	−1.390	−1.394	−1.293
	LOC_Os04g19740	Transketolase	3.525	3.068	2.905	3.630	3.341
	LOC_Os03g16050	phosphoric ester hydrolase	−2.582	−2.252	−2.539	−1.504	−1.300
	LOC_Os01g64660	phosphoric ester hydrolase	−3.897	−3.802	−4.389	−3.268	−2.591
	LOC_Os11g07020	fructose-bisphospate aldolase isozyme	−3.383	−2.558	−2.670	−2.045	−2.522
	LOC_Os06g40640	fructose-bisphosphate aldolase	−2.820	−2.738	−3.224	−2.088	−1.927
	LOC_Os01g02880	fructose-bisphosphate aldolase	1.500	1.333	1.412	1.480	1.342
	LOC_Os10g08960	pyridoxin biosynthesis protein ER1, putative	−1.946	−1.623	−1.534	−1.371	−1.176
	LOC_Os10g30550	tRNA methyltransferase, putative, expressed	1.974	2.427	1.890	2.048	2.217
	LOC_Os06g45710	phosphoglycerate kinase	3.423	3.643	3.260	3.662	3.066
	LOC_Os05g41640	phosphoglycerate kinase	−3.760	−3.652	−4.003	−3.513	−3.312
	LOC_Os03g19240	AMP-binding enzyme, putative, expressed	−4.206	−4.022	−3.931	−3.537	−3.428
	LOC_Os03g51740	tyrosine transaminase	−3.771	−5.202	−5.041	−4.316	−4.475
	LOC_Os11g02760	ribulose-bisphosphate carboxylase	−3.068	−3.428	−2.905	−3.534	−2.015
	LOC_Os03g09090	ribulose-bisphosphate carboxylase	1.578	1.350	1.501	1.521	1.820
	LOC_Os12g17600	ribulose-bisphosphate carboxylase	−5.512	−5.732	−6.115	−4.999	−5.034
	LOC_Os12g19381	ribulose-bisphosphate carboxylase	−3.091	−3.135	−3.337	−2.661	−3.216
	LOC_Os12g19394	ribulose-bisphosphate carboxylase	−4.124	−4.089	−4.936	−3.427	−3.177
	LOC_Os12g19470	ribulose-bisphosphate carboxylase	−3.197	−2.578	−3.097	−2.215	−1.978
	LOC_Os11g32770	ribulose bisphosphate carboxylase large chain precursor	−2.279	−2.726	−2.444	−1.093	−1.093
	LOC_Os11g47970	ribulose bisphosphate carboxylase/oxygenase activase, chloroplast precursor	−4.174	−3.598	−3.109	−2.294	−2.931
	LOC_Os10g21280	ribulose bisphosphate carboxylase large chain precursor	−2.597	−2.746	−2.203	−1.288	−1.083
Photorespiration	LOC_Os03g52840	glycine hydroxymethyltransferase	−3.369	−3.170	−3.199	−2.666	−2.509
	LOC_Os08g37940	phosphoglycolate phosphatase	−3.938	−3.938	−4.637	−4.789	−4.146
	LOC_Os04g41340	phosphoglycolate phosphatase	−3.807	−3.432	−3.621	−2.600	−2.386
	LOC_Os03g36750	phosphoglycolate phosphatase	−2.712	−2.462	−2.400	−2.516	−2.328
	LOC_Os03g19760	phosphoglycolate phosphatase	−1.144	−1.076	−1.134	−1.253	−1.167
	LOC_Os02g57100	Hydrolase	−2.129	−1.749	−1.795	−2.283	−1.990
** Antenna proteins	LOC_Os01g41710		−6.434	−6.434	−6.409	−5.622	−6.342
	LOC_Os01g52240		−6.346	−6.441	−5.927	−6.023	−5.902
	LOC_Os02g10390	Light-harvesting chlorophyll-protein complex	−1.972	−1.823	−2.190	−1.020	−1.958
	LOC_Os02g52650	Light-harvesting chlorophyll-protein complex	−2.998	−2.967	−3.451	−3.194	−2.554
	LOC_Os03g39610		−3.766	−3.797	−4.078	−3.380	−3.969
	LOC_Os04g38410		−5.128	−4.972	−5.069	−5.043	−5.131
	LOC_Os06g21590	Light-harvesting chlorophyll-protein complex	−2.223	−2.249	−2.761	−1.919	−2.098
	LOC_Os07g38960	Light-harvesting chlorophyll-protein complex	−3.290	−3.182	−3.243	−2.598	−2.337
	LOC_Os08g33820	Light-harvesting chlorophyll-protein complex	−2.651	−2.651	−2.999	−2.582	−2.504
	LOC_Os07g37240	Light-harvesting chlorophyll-protein complex	−5.463	−5.674	−5.660	−4.875	−4.614
	LOC_Os09g17740		−2.365	−1.729	−1.918	−1.438	−1.943
	LOC_Os07g37550	Light-harvesting chlorophyll-protein complex	−2.005	−1.726	−2.079	−1.022	−1.637
	LOC_Os11g13890	Light-harvesting chlorophyll-protein complex	−4.353	−4.353	−4.858	−3.783	−3.739
Photosystem and electron transport system	LOC_Os02g51470	F-type ATPase	−1.708	−1.475	−1.876	−1.781	−1.645
	LOC_Os07g32880	F-type ATPase	−2.999	−2.940	−2.441	−2.189	−2.222
	LOC_Os02g01340	Photosynthetic electron transport	−1.446	−1.317	−1.640	−1.011	−1.184
	LOC_Os03g48040	Photosynthetic electron transport	−2.058	−1.945	−2.137	−1.937	−1.935
	LOC_Os06g01210	Photosynthetic electron transport	−3.000	−2.915	−3.315	−2.440	−3.101
	LOC_Os06g01850	Photosynthetic electron transport	−2.085	−1.803	−2.114	−1.728	−1.800
	LOC_Os08g01380	Photosynthetic electron transport	−3.417	−2.474	−2.750	−2.516	−2.461
	LOC_Os03g56670	Photosystem I (P700 chlorophyll a)	−2.801	−2.714	−2.990	−2.314	−2.535
	LOC_Os05g48630	Photosystem I (P700 chlorophyll a)	−1.841	−1.810	−1.876	−1.990	−1.596
	LOC_Os07g05480	Photosystem I (P700 chlorophyll a)	−4.478	−4.499	−5.141	−4.467	−4.418
	LOC_Os07g25430	Photosystem I (P700 chlorophyll a)	−2.573	−2.470	−2.949	−2.652	−2.617
	LOC_Os08g44680	Photosystem I (P700 chlorophyll a)	−2.151	−2.231	−2.468	−1.649	−2.432
	LOC_Os09g30340	Photosystem I (P700 chlorophyll a)	−4.405	−4.523	−4.857	−3.808	−3.681
	LOC_Os12g08770	Photosystem I (P700 chlorophyll a)	−4.552	−4.540	−4.975	−3.700	−4.317
	LOC_Os12g23200	Photosystem I (P700 chlorophyll a)	−2.255	−1.965	−2.449	−1.349	−1.924
	LOC_Os01g31690	Photosystem II (P680 chlorophyll a)	−3.108	−3.089	−2.917	−2.665	−2.833
	LOC_Os01g56680	Photosystem II (P680 chlorophyll a)	−3.117	−3.034	−3.817	−3.196	−3.728
	LOC_Os01g64960	Photosystem II (P680 chlorophyll a)	−2.733	−2.504	−3.035	−2.133	−2.002
	LOC_Os01g71190	Photosystem II (P680 chlorophyll a)	−2.982	−2.887	−3.073	−2.683	−2.349
	LOC_Os03g21560	Photosystem II (P680 chlorophyll a)	−2.878	−2.814	−3.727	−3.547	−3.263
	LOC_Os07g04840	Photosystem II (P680 chlorophyll a)	−3.009	−2.943	−3.494	−2.656	−2.836
	LOC_Os07g36080	Photosystem II (P680 chlorophyll a)	−5.421	−5.449	−5.847	−4.821	−4.372
	LOC_Os08g02630	Photosystem II (P680 chlorophyll a)	−3.645	−3.537	−3.488	−2.570	−2.621
Xanthophyll_Cycle	LOC_Os04g31040	violaxanthin de-epoxidase	−2.836	−2.562	−2.680	−2.452	−2.284
	LOC_Os01g51860	violaxanthin de-epoxidase	−1.326	−1.326	−1.479	−1.290	−1.198
chlorophyll a biosynthesis II	LOC_Os02g51080	geranylgeranyl reductase	−2.447	−2.447	−2.803	−1.883	−2.140
	LOC_Os02g51080	geranylgeranyl reductase	−2.447	−2.447	−2.803	−1.883	−2.140

**†** Rice NILs: NIL10 = IR77298-14-1-2-B-10; NIL13 = IR77298-14-1-2-B-13, NIL18 = IR77298-5-6-B-18; NIL11 = IR77298-5-6-B-11; * Signal intensities of gene expression from microarray data (4 × 44 K, agilenttechnologies) which converted to log2; Negative log2ratio are down-regulated genes and positive (yellow) log_2_ratio are up-regulated genes; Genes with no change in their expression are not shown; Severe drought stress: water deficit treatments with a fraction of transpirable soil water (FTSW) of 20 percent.

Drought stress in various plants has also been studied using proteomics techniques. Most of the leaf proteome analysis in response to water stress clearly confirmed the regulation of the proteins related to photosynthesis pathway. The activity of enzymes related to photoelectron transport and carbon reduction cycle, including the key enzyme RuBisCO are reduced under drought stress [50,51,52]. Regulation of this protein is highly affected by the duration and severity of the stress as well as plant type. Summary of selected proteins involved in photosynthesis pathway in response to abiotic stress is represented in Table 2. Up-regulation of chloroplast ATP synthase, both the CF1α and CF1β [52,53], cytochrome *b_6_*/*f* complex, chloroplast oxygen-evolving enhancer protein 1 [54,55], two key enzymes involved in sucrose utilisation, invertase and sucrose synthase [56] were reported in response to drought stress. However, the activity of the key enzyme in sucrose synthesis, sucrose-phosphate synthase, is down-regulated by water stress [56].

**Table 2:** Summary of selected differentially expressed proteins involved in photosynthesis pathway in response to abiotic stress.

Stress	Plant Species	Protein Description	Expression *	Ref.
Drought	Sugar cane (*Saccharum officinarum* L.)	phosphoenolpyruvate carboxylase; NADP malic enzyme; pyruvate orthophosphate dikinase	−	[4]
	Maize (*Zea mays* L.)	phosphoenolpyruvate carboxylase	−	[4,27]
	Arabidopsis (*Arabidopsis thaliana*)	fructose-1,6-bisphosphatase; genes related to ATP synthesis, PSI and PSII	−	[20]
	Norway spruce (*Piceaabies*)Wheat (*Triticum aestivum* L.)	oxygen-evolving enhancer protein 2 RuBisCO LS	+	[55,57]
	Cotton (*Gossypium herbaceum*)	RuBisCO subunit binding protein	−	[52]
	Cotton (*Gossypium herbaceum*)	chloroplast ATP synthase	+	[52]
	Wheat (*Triticum aestivum* L.)	RuBisCOactivase; RuBisCO LS	+	
	Wild watermelon (*Citrullus lanatus*)	ATP synthase; chloroplast Rieske ISP; RuBisCO SS; PSI subunit D; oxygen-evolving enhancer protein 2	+	
Salinity	Common bean (*Phaseolus vulgaris* L.); Sunflower (*Helianthus annuus* L.); Wheat (*Triticum aestivum* L.); Maize (*Zea mays* L.)	fructose-1,6-bisphosphatase; RuBisCO; phosphoenolpyruvate carboxylase; ATP synthase	−	[4,54,58]
	Potato (*Solanum tuberosum* L.); Rice (*Oryza sativa* L.)	fructose-1,6-bisphosphatase	+	[4]
	Arabidopsis (*Arabidopsis thaliana*)	fructose-1,6-bisphosphatase; genes related to ATP synthesis, PSI and PSII	−	[20]
	Wheat (*Triticum aestivum* L.)	oxygen-evolving enhancer protein 2; RuBisCOactivase;	+	[54]
	Maize (*Zea mays* L.)	23 kDa polypeptides of PSII; ferredoxin NADPH1; oxidoreductase; chlorophyll a/b binding protein	+	[58]
Heat	Maize (*Zea mays* L.)	RuBisCOactivase	−	[4]
	Cotton (*Gossypium hirsutum* L.); Tobacco (*Nicotiana tabacum* L.)	RuBisCO	−	[4]
	Wheat (*Triticum aestivum* L.)	RuBisCO; phosphoenolpyruvate carboxylase	−	[4]
	*Populus euphratica*	PSII stability/assembly factor	−	[59]
	*Populus euphratica*	plastid ATP synthase CF1 a chain	+	[59]
	Rice (*Oryza sativa* L.)	glyceraldehyde-3-phosphate dehydrogenase; RuBisCO LS; 23 kDa polypeptide of PSII; Oxygen-evolving complex protein 1; oxygen-evolving protein of PSII;	−	[60]
	Rice (*Oryza sativa* L.)	RuBisCOactivase; glutamine synthetase; glyceraldehyde-3-phosphate dehydrogenase;	+	[60]
Flooding	Cacao (*Theobroma cacao*)	oxygen-evolving enhancer protein; light harvesting chlorophyll a/b-binding protein; light-harvesting complex II protein Lhcb2; light-harvesting complex I chlorophyll a/b binding protein 3; phosphate dikinase 1	+	[61]
Cold	*Thellungiella halophila*	glyceraldehyde-3-phosphate dehydrogenase; oxygen-evolving enhancer 33; RuBisCO SS	+	[62]
	Maize (*Zea mays* L.)	cytochrome b561/ferric reductase	−	[62]

* Up- and down-regulation are represented as + and −, respectively.

Not only the leaf, but also the root proteome is also highly affected by drought stress which subsequently impairs photosynthesis in plants. Two key enzymes of carbohydrate metabolism, UDP-glucose pyrophosphorylase and 2,3-bisphosphoglycerate independent phosphoglycerate mutase, were down-regulated in soybean root upon exposure to drought [63]. The levels of abundance of both proteins tended to revert to that of the control plants when watering was restored. Because the shift in carbon partitioning under drought stress is an adaptive response, a decrease in the expression of glycolytic enzymes in response to drought stress might be a consequence of reduced growth. Furthermore, it is a mechanism for accumulating sugars as an energy source for recovery and rapid growth once water is available. It has been shown that the expression of *S*-adenosylmethionine synthetase in soybean root decreases upon exposure to drought stress [63]. Down-regulation of this enzyme under drought is consistent with the inhibition of photosynthetic activity as a general feature of abiotic stresses. It is postulated that whole plant should be considered to study of photosynthesis activity in response to drought stress.

### 3.2. Salinity

Salt stress derived from the high accumulation of salts near the root zone causes accumulation of saline ions in plant tissues. Osmotic effect and ion toxicity are two main reasons of growth reduction, when plants are exposed to salt stress. Although several reports did not separate these two effects, Munns [64] proposed a two-phase growth model in the plant response to salt. According to the model, water deficit is the primary effect of salt stress (Phase 1) with equal effect on leaf expansion rate of a given plant species regardless of the degree of tolerance. The effect of ions (Phase 2) causes the leaves of a sensitive variety to die faster. Restriction of CO_2_ diffusion into the chloroplast and reduction of carbon metabolism are among the physiological changes in leaves under salt stress [20]. Intensity and duration of the stress, leaf age as well as plant species are the main determining factors in plant response to the stress.

It is crucial to determine the molecular basis of the variation in important traits such as photosynthesis rate in plants in response to salinity stress. Photosynthesis is a complex pathway and many genes are involved in this system, hence when plants are exposed to salinity stress, expression of several photosynthesis related genes may change. Chaves *et al.* [20] summarized the number of affected genes and proteins of some model plants under drought and salt stress. They showed that several genes related to ATP synthase, PSI and PSII were down-regulated by salt and drought stress. In-depth study of the mitochondrial proteome during salt stress induced programmed cell death in rice was performed by Chen *et al.* [65]. *S*-adenosylmethionine synthetase was among the four down-regulated proteins in response to salt stress. This protein was already discussed to be down-regulated in response to drought stress.

Photosynthesis-related proteins were down-regulated in soybean seedling leaf under salt stress [66]. Sobhanian *et al.* [67], reviewed the effects of salt stress on several plants, including rice, soybean, wheat, potato and *Aleuropus lagopoides*. They concluded that reducing photosynthesis activity under salt stress was the only common response in the plants. Using *in vivo* hydroponic rice seedling culture system, proteome of rice leaves under salt stress was evaluated. Among the photosynthesis related proteins, oxygen evolution proteins, a protein related to PSII, was up-regulated in response to salt stress [68]. Salt stress alters the expression of proteins even after hours of stress exposure. It has been shown that in the initial phase of moderate salt stress (up to 4 h), sodium ions accumulate quickly and excessively in chloroplast of maize. This could enhance the expression of polypeptides of PSII, ferredoxin NADPH^+^ oxidoreductase, ATP synthase and chlorophyll a/bbinding protein [58]. Since the water potential of the leaves remained unchanged, it can be assumed that the rapid response of plant to salinity, is a reflecting mechanism to alleviate the detrimental effects of sodium ions on the photosynthetic machinery.

### 3.3. Cold

Cold climate significantly reduces crop productivity and when the temperature drops to the freezing point, the damage is more severe. The key change in plant cells when exposed to cold stress is the fluidity of the membrane and by reduction of the fluidity, the plant cell senses cold stress [69]. Changes in carbohydrate metabolism, secondary metabolism and photosynthesis are other common responses of plant cells under cold stress. It has been shown that cold stress significantly altered the maximum quantum yield of PSII (Fv/Fm), the maximum photo-oxidizable P700 (Pm), the energy distribution in PSII and the redox state of P700 in seedlings of three promising oilseed crops originating from tropical regions [70]. Although PSI is the main target of stress under cold conditions, it has been shown that PSII is more sensitive to low temperature than PSI [70,71]. A decrease of photosynthetic activity, may cause photodamage which ultimately causes generation of ROS. Aggregation of ROS may inhibit protein synthesis, necessary for the repair of photodamage. The D1 protein, required for the repair of PSII, is one of the proteins suppressed by ROS [70].

**Figure 1:**
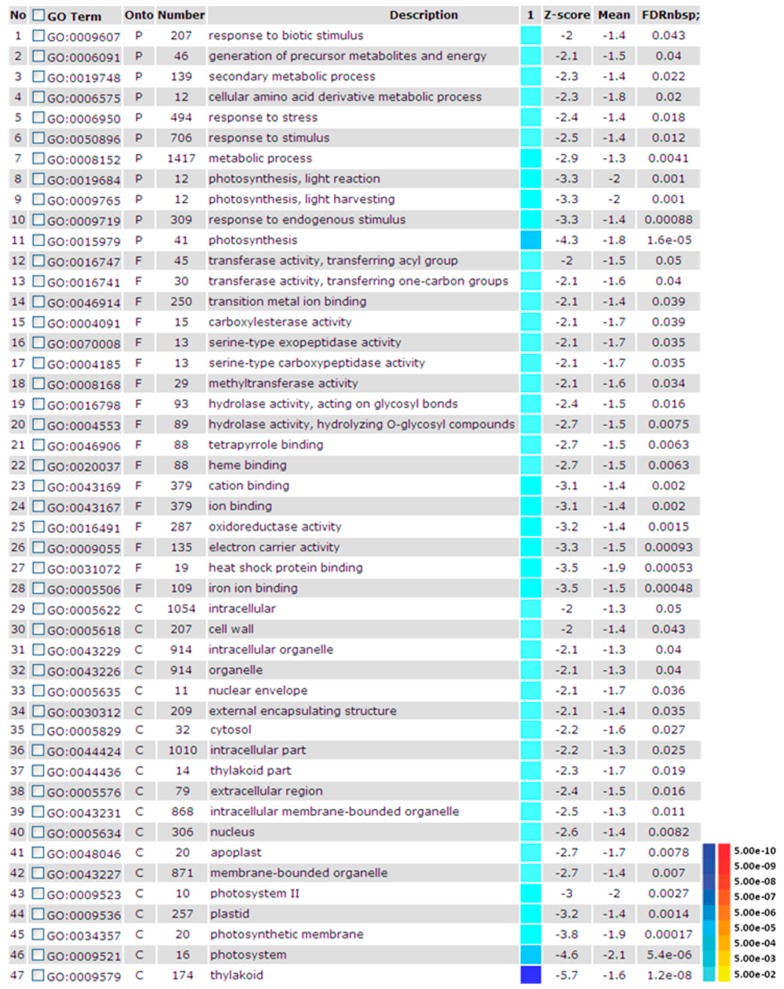
Gene ontology (GO) analysis of the down-regulated differentially expressed genes of rice in response to cold treatment. This figure shows a colorful model of the PAGE analysis of gene expression data under the cold treatment after 72 h. The information includes the following: GO terms, (including 3 GO categories: biological process (P), molecular function (F) and cellular component (C)), number of annotated genes for each GO term, GO description, a simple colorful model in which the red color system indicates up-regulation and blue color indicates down-regulation, and different statistical parameters such as z-scores, means and adjusted P values (FDR) in the different rice genotypes.

In rice, as a model C_3_ plant, several reports indicated that cold stress prevents chlorophyll synthesis and chloroplast formation in leaf tissues. Therefore, a reduction in chlorophyll content can be a sign of low temperature effect on rice genotypes [72]. Previous study showed that when rice seedling were treated at 10 °C for 72 h, a large number of genes including those involved in photosynthesis were highly down-regulated (Figure 1). Proteins related to the photosynthetic pathway are widely affected by low temperature stress. In C_4_ plants, however, cold stress is one of the main limiting factors for growth and development. Although there are examples of C_4_ species with cold adaptation, they cannot compete with C_3_ plants in cold climates. Sage and McKown [73] noted possible reasons of poor C_4_ photosynthesis rate at cold climate which are: declinein activity of the C_4_-cycle enzymes phosphoenolpyruvate carboxylase and pyruvate phosphate dikinase; lower maximum quantum yield of C_4_ photosynthesis compared to C_3_ species in low temperature environment; and limitation in RuBisCO capacity [73]. Thus, the effect of cold on the photosynthesis of C_4_ plants is more severe than C_3_ plants.

Gao *et al.* [62] analyzed the proteome of *Thellungiella halophyila*, a chilling-tolerant plant, under cold stress and reported that 28% of the regulated proteins were photosynthesis-related proteins. Glyceraldehydes-3-phosphate dehydrogenase B, chloroplast precursor, RuBisCO small and large subunits, chloroplast carbonic anhydrase precursor andpastocyanin, oxygen-evolving enhancer, cytochrome *b_6_*/*f* complex iron- sulfur subunit, and alanine-2-oxoglutarate aminotransferase were among the regulated proteins. Identification of a large number of chloroplast-related proteins (nearly half of the regulated proteins) supports the idea that cold stress tolerance of *T. halophila*is achieved, at least partly, by regulation of chloroplast function.

### 3.4. Light

The photosynthesis system in plants is directly related to both quality and quantity of light. Changes in light intensity leads to imbalance in light captue by the photosystems. To ensure optimal photosynthesis efficiency, plants adjust the relative abundance of PSI and PSII according to the light quality [9]. When plants are exposed to high light intensity, a rapid repression may happen in genes encoding light-harvesting complex components, PSI and PSII reaction centre subunits [74,75]. While PSII is highly susceptible to photodamage, PSI is efficiently protected against photodamage. However, photoinhibition of PSI has been reported similar to PSII in Arabidopsis when plants are exposed to low temperature [76]. Foyer *et al.* [9], explained the mechanisms that regulate reactions in the photosynthetic electron transport chain so that the rate of production of ATP and NADPH is coordinated with the rate of their utilization in metabolism. This mechanism optimizes light use efficiency at low irradiance or dissipates excess excitation energy as heat at high light condition. The energy absorbed by plants under high irradiance exceeds the capacity of light utilization in photosynthesis and this cause photoinhibition. Although PSII is a primary site of inhibition, there is evidence that under certain circumstances, PSI can be photoinhibited even faster than PSII [77]. According to a review of the literature, most of the studies focused on high light stress rather than low light irradiance, because of the deletorious effects of photoinhibition and photodamage on plants [76,77].

It has been shown that transcripts encoding proteins involved in photoprotection such as the PSII-S and early light inducible protein 2 (ELIP2) are enhanced in high light [74,75]. ELIPs are nuclear-encoded proteins belonging to the chlorophyll a/b-binding protein family located in thylakoid membranes. The proposed function of the protein is a transient binding to the released chlorophylls under high light stress and prevent the formation of free radicals [78]. Recently, a proteome analysis of *Arabidopsis* in response to increased light conditions could identify proteins related to photosynthesis, carbon metabolism and plastid mRNA processing. The results confirmed the participation of the EXECUTER proteins in signalling and control of chloroplast metabolism, and in the regulation of plant response to environmental changes [79]. A proteome analysis of *Arabidopsis* exposed to high light conditions revealed that 35 out of 64 identified proteins were related to photosynthesis [80]. Therefore, it can be assumed that the deletorius effects of light stress on the photosynthetic pathway is higher than otherabiotic stresses.

### 3.5. Flooding

Flooding is a complex abiotic stress that affects the growth and development of plants and significantly decreases the productivity of crops. Higher plants are aerobic organisms that die when oxygen availability is limited due to soil flooding [81]. Plants may encounter oxygen depletion as a preliminary stress signal, when the soil water content rises above field capacity [69]. The response of plant to flooding stress is highly correlated with the level of tolerance or susceptibility to the stress. It has been shown that reduction in gas exchange parameters was varied among the citrus genotypes, and the tolerant genotypes maintain CO_2_ assimilation rate and carboxylative efficiency at control levels for a longer time than sensitive genotypes under continuously flooded conditions [82]. Thus, the ability to maintain gas exchange parameters may be presumed as the main indication of tolerance to flooding. Mutava *et al.* [83] used four contrasting soybean genotypes for tolerance or susceptibility to flooding and drought. They reported different mechanisms contributing to the reduction of net photosynthesis under drought and flooding. Under drought stress, ABA and stomatal conductance were responsible for reduced photosynthetic rate; while under flooding stress, accumulation of starch granules played a major role.

Transcriptional responses to flooding stress in soybean seedlings have been studied by Nanjo *et al.* [84]. Using a soybean microarray chip, genome-wide changes in gene expression were analyzed in which photosynthsis related genes were up-regulated [84]. A comparison of soybean tolerant and susceptible genotypes showed that seven fibrillin proteins were up-regulated in the tolerant genotype, but down-regulated in the susceptible genotype [83]. Photosynthesis related proteins can be identified not only in the leaf, but also in other green organs. A comparative proteomic analysis of cotyledon of soybean under flooding stress using gel-free quantitative techniques resulted in the identification of 165 proteins which are commonly observed in both control and flooding-stressed plants. Photosynthesis related proteins were not among the main group of the regulated proteins. However, the role of ferritin in protecting plant cells against oxidative damage under flooding conditions was highlighted [85]. Study of gene expression and protein profiles of clonal genotypes of cocoa in response to flooding stress indicated that photosynthesis-associated proteins such asoxygen-evolving enhancer protein, light harvesting chlorophyll a/b binding protein, RuBisCO activase, light harvesting complex I and complex II proteins were highly up-regulated [61]. These proteins improve the plant’s ability to maintain glycolysis and induce fermentation against anoxia and may also serve to distinguish tolerant and susceptible genotypes.

### 3.6. Heat

Physiological studies have indicated that short and long-term exposure to heat stress in temperate and tropical crops reduced the net photosynthesis rate and the PSII activity [86]. High temperature can significantly reducethe maximal quantum yield of PSII in plants [87] such as rice [60,88], *Populus* [59] and tomato [89]. Chloroplast and thylakoid membrane are highly susceptible to heat stress. Carbon metabolism of the stroma, photochemical reactions in thylakoid lamellae and changes instructural organization of thylakoids are considered as the primary sites of injury in chloroplast at heat stress [87,90]. It also caused reduction in electron transport, damage to photosystems and activation of the glycolate pathway and generation of H_2_O_2_ in poplar [91].

Hasanuzzaman *et al.* [87] reviewed the molecular mechanisms of high temperature stress in different crop species. Reduction inthe amount of photosynthetic pigments as a result of lipid peroxidation of chloroplast and thylakoid membranes, decrease in gas exchange and CO_2_ assimilation rates, leaf water potential, leaf stomatal conductance, intercellular CO_2_ concentration, total chlorophyll content and leaf area were major effects of heat stress in plants. Heat stress reduces starch and sucrose synthesis, soluble proteins, RuBisCO binding proteins and large and small subunits. It also decreases the activity of sucrose phosphate synthase, ADP-glucose pyrophosphorylase, and invertase [87].

Gene ontology analysis under moderately high temperatures (30 °C) revealed a significant increase in the expression of transcripts related to photosynthesis and those encoding polypeptides associated with PSII, ferredoxins, subunits of RuBisCO, RuBisCO activase and a number of Calvin cycle enzymes [92]. It seems that under moderate high temperature, plants may improve the photosynthesis mechanisms aimed at reaching the best possible performance in the new situation. However, when plant encounter heat stress, the expression of a wide range of proteins are down-regulated. Proteins associated with primary carbon assimilation, Calvin cycles, PSI, PSII, RuBisCO subunits, carbonic anhydrase, electron transport proteins such as oxygen-evolving enhancer protein and ferredoxin-NADP reductase are down-regulated following exposure to heat [60,86]. The opposite reaction of plants under acute heat stress, indicates that the photosynthesis apparatus is impaired by the stress.

## 4. Nucleus and Chloroplat Genes/Proteins Control Photosynthesis under Abiotic Stress

### 4.1. Coordination of Nucleus and Chloroplast

Photosynthesis is a complex mechanism in green plants as a result of a coordination between chloroplast and other organelles and compartments of the cell. Chloroplasts are the main site for light- and dark-dependent reactions of photosynthesis. Besides photoassimilation of carbohydrates, chloroplasts are involved in the biosynthesis of lipids, aromatic amino acids, hormones, vitamins, and secondary metabolites [93]. This organelle is highly sensitive to various environmental stresses and it is one of the initial sites of the stress response in plant cells [3]. Under stress conditions, electron balance and redox homeostasis are highly important. Therefore, proteins involved in electron transport play a major role in the chloroplast. Ferredoxins are small and soluble proteins that play a key role in electron distribution in all types of plastids [94]. Down-regulation of ferredoxin, shortage of NADP^+^and over-reduction of the photosynthetic electron transport chain forms superoxide radicals and singlet oxygen in the chloroplast.

In the process of photosynthesis, the chloroplast and nucleus regulates almost all of the required genes and proteins in photosynthesis. The number of proteins in chloroplasts which are encoded in the nucleus is significantly higher than that in plastid. It has been reported that chloroplasts contain 3000–4000 different proteins which are mostly encoded in the nucleus, with only a small number encoded in the plastid genome [95]. According to an estimation, in comparison with about 2300 nuclear-encoded proteins in the chloroplast of Arabidopsis, only 87 proteins are plastid-encoded [96]. Therefore, a high degree of coordination should exist between nucleus and chloroplast to express the required genes. The mechanisms of coordination and bilateral information exchange between nucleus and chloroplast are explained by Fey *et al.* [95], where the role of redox signals as well as photosynthetic products like sugars and ROS as photosynthetic by-products in plastid-to-nucleus signaling are highlighted. The signaling components such as genomes uncoupled 1, cryptochrome1 and chloroplastic EXECUTER proteins which mediate signaling processes to the nucleus were explained by Kangasjärvi *et al.* [93]. Recently, various mechanisms involved in the signals coming from the chloroplasts to the nucleus via retrograde signaling were explained [97]; the nucleus-plastid coordination and signaling mechanisms are more important when a plant is exposed to abiotic stress since this coordination should lead to the alleviation of damage to the photosynthesis system of plants.

### 4.2. Overexpression of Photosynthesis-Related Proteins

The performance of photosynthetic pathways is improved in transgenic plants overexpressing the genes encoding photosynthesis-related proteins under abiotic stress. It has been shown that co-expression of the two soluble flavoproteins in the chloroplast stroma reduced ROS accumulation and improved the tolerance to stress [98]. Overexpression of MYB transcription factor in *Arabidopsis* [99]; glycine betaine in rice and tomato [100,101]; betaine aldehyde dehydrogenase in sweet potato [102]; plastidal protein synthesis elongation factor in wheat [103]; chloroplast small heat shock protein in tomato [104]; sucrose non-fermenting1-related protein kinase 2 in *Arabidopsis* [105]; Na^+^/H^+^ antiporter in *Arabidopsis* and cotton [106,107] and aquaporin in tobacco [108] are already reported. Transgenic plants with the overexpressed genes performed at least one of the following characteristics under abiotic stress conditions: enhance cell membrane stability, CO_2_ fixation rate, PSII activity and photosynthetic rate, and reduce heat injury to thylakoids, electrolyte leakage and destruction of chlorophyll. To have efficient protein overexpression, understanding the type of the stress and its effects on plant is necessary. A survey on the localization of the overexpressed proteins related to photosynthesis pathway indicated that not all of the proteins are localized in the chloroplast [16]. Abundance of proteinin other organelles indicates the complexity of the pathway and highlights the role of signaling especially under abiotic stress.

### 4.3. Role of Thylakoid Membrane in Photosynthesis

A thylakoid, a membrane-bound compartment inside chloroplasts, is a place for primary reactions of photosynthesis. The thylakoid membrane contains the four major multisubunit protein complexes, PSI, PSII, ATP synthase complex and cytochrome *b_6_*/*f* complex. Around 100 proteins are controlling the reactions in thylakoid membranes. The proteins are mainly involved in the conversion of light energy to chemical energy, but several other proteins have a function in assembly, maintenance, and regulation of the four multiprotein complexes [17]. Isolation and analysis of proteins located in thylakoid membrane can give an overview of the complex function of the membrane especially under stress conditions. Thylakoid membrane proteins were isolated from wild-type and mutant strains of *Chlamydomonas reinhardtii* and analyzed using proteomic techniques. More than 30 different spots were identified as light-harvesting complex proteins. The function of this protein in response to abiotic stress was reported in plants by Kono *et al.* [109] and Muneer *et al.* [110].

Environmental stress impairs the activity of the thylakoid membrane by disruption of the membrane, thereby, inhibiting the activities of membrane-associated electron carriers and enzymes and resulting in reduction of the PSI and PSII and photosynthesis rate [3]. Fluctuation of light intensity affects the architecture and protein distribution of thylakoids. It has been shown that chloroplasts in the shade havea higher density of thylakoids per chloroplast sectional area and more extensive grana stacks, and thereby more granal thylakoids than chloroplasts in the sun [109] indicating variation in the capacity of photosynthetic electron transport.

## 5. Phosphorylation of the Photosynthesis-Related Proteins under Abiotic Stress

Protein posphorylation at specific serine, threonine and tyrosine residues is able to change many properties of proteins such as interaction with other proteins, stability, localization and activity [111]. Reversible phosphorylation plays an important role in the regulation of cellular mechanism, signaling pathways and several developmental processes of plant such as cell growth, differentiation, migration, metabolism, apoptosis and stress responses [112,113]. Under abiotic stress, reversible protein phosphorylation is a powerful tool to alleviate damage to plant cell and specifically photosynthesis system. Phosphorylation of the thylakoid proteins in response to drought, high light, cold, heat and nutrient deficiency was reported; phosphorylation sites in thylakoid proteins from the green alga exposed to different environmental conditions indicated that 31 *in vivo* protein phosphorylation sites affect the photosynthetic machinery in the alga [111]. A survey of the photosynthetic pathways indicated that several critical functions of photosynthesis-related proteins are under control of reversible phosphorylation. The reversible phosphorylation of chlorophyll a/b binding proteins is part of the light-harvesting complex under stress for balancing the excitation energy between the PSI and PSII [109,114]. PSII core protein, the D1, D2, and CP43 phosphorylation in the photoinhibition-repair cycle [115] are known to be involved in photosynthesis pathways. Although, phosphorylation and dephosphorylation of the photosynthesis-related proteins occur under ambient conditions, the role of the modification in reduction of damage is of greater significance under stress conditions.

## 6. Conclusions and Future Perspectives

Environmental stresses are great challenges for the growth and development of plants. When plants are exposed to abiotic stress, photosynthetic pathways are highly affected. In crops, reduction in photosynthesis rate, significantly decrease assimilates and ultimately reduce the yield. Response to the specific stress is highly dependent on the level of tolerance or susceptibility of plants to the stress which is mostly controlled by the expression of nuclear genes and proteins. Nuclear gene expression under stress condition is controlled by retrograde signaling pathways. The signals can regulate the expression of genes which leads to the expression of proteins. There are four major multi-subunit protein complexes in the process of photosynthesis. Although all of the complexes are affected by abiotic stress, review of the regulated proteins under stresses highlights a significant role for PSII in the thylakoid membrane. Therefore, more attention should be paid to the photosynthesis system, especially to PSII, when the target is the production of an abiotic stress tolerant plant. Furthermore, because of the coordinated mechanisms in photosynthetic pathways, the whole plant should be considered to alleviate the deleterious effects of the abiotic stress.

## References

[1] Gupta B., Sengupta A., Saha J., Gupta K. (2013). Plant abiotic stress: ‘Omics’ approach. J. Plant Biochem. Physiol..

[2] Miranda H. (2011). Stress Response in Cyanobacterium *Synechocystis* sp. PCC 6803. Ph.D. Thesis.

[3] Pinheiro C., Chaves M.M. (2011). Photosynthesis and drought: Can we make metabolic connections from available data?. J. Exp. Bot..

[4] Ashraf M., Harris P.J.C. (2013). Photosynthesis under stressful environments: An overview. Photosynthetica.

[5] Komatsu S., Hossain Z. (2013). Organ-specific proteome analysis for identification of abiotic stress response mechanism in crop. Front. Plant Sci..

[6] Gunawardana D. (2008). Supercharging the rice engine. Rice Today.

[7] Pego J.V., Kortstee A.J., Huijser C., Smeekens S.C.M. (2000). Photosynthesis, sugars and the regulation of gene expression. J. Exp. Bot..

[8] Eberhard S., Finazzi G., Wollman F.A. (2008). The dynamics of photosynthesis. Annu. Rev. Genet..

[9] Foyer C.H., Neukermans J., Queval G., Noctor G., Harbinson J. (2012). Photosynthetic control of electron transport and the regulation of gene expression. J. Exp. Bot..

[10] Berry J.O., Yerramsetty P., Zielinski A.M., Mure C.M. (2013). Photosynthetic gene expression in higher plants. Photosynth. Res..

[11] Ambavaram M.M.R., Basu S., Krishnan A., Ramegowda V., Batlang U., Rahman L., Baisakh N., Pereira A. (2014). Coordinated regulation of photosynthesis in rice increases yield and tolerance to environmental stress. Nat. Commun..

[12] Maayan I., Shaya F., Ratner K., Mani Y., Lavee S., Avidan B., Shahak Y., Ostersetzer-Biran O. (2008). Photosynthetic activity during olive (*Olea europaea*) leaf development correlates with plastid biogenesis and RuBisCO levels. Physiol. Plant..

[13] Urban O., Sprtova M., Kosvancova M., Tomaskova I., Lichtenthaler H.K., Marek M.V. (2008). Comparison of photosynthetic induction and transient limitations during the induction phase in young and mature leaves from three poplar clones. Tree Physiol..

[14] Woodson J.D., Chory J. (2008). Coordination of gene expression between organellar and nuclear genomes. Nat. Rev. Genet..

[15] Blankenburg M., Haberland L., Elvers H.D., Tannert C., Jandrig B. (2009). High-throughput omics technologies: Potential tools for the investigation of influences of EMF on biological systems. Curr. Genom..

[16] Nouri M.Z., Komatsu S. (2013). Subcellular protein overexpression to develop abiotic stress tolerant plants. Front. Plant Sci..

[17] Hippler M., Klein J., Fink A., Allinger T., Hoerth P. (2001). Towards functional proteomics of membrane protein complexes: Analysis of thylakoid membranes from *Chlamydomonas reinhardtii*. Plant J..

[18] Taiz L., Zeiger E. (2010). Plant Physiology.

[19] Ahuja I., de Vos R.C., Bones A.M., Hall R.D. (2010). Plant molecular stress responses face climate change. Trends Plant Sci..

[20] Chaves M.M., Flexas J., Pinheiro C. (2009). Photosynthesis under drought and salt stress: regulation mechanisms from whole plant to cell. Ann. Bot..

[21] Prins A., Mukubi J.M., Pellny T.K., Verrier P.J., Beyene G., Lopes M.S., Emami K., Treumann A., Lelarge-Trouverie C., Noctor G. (2011). Acclimation to high CO_2_ in maize is related to water status and dependent on leaf rank. Plant Cell Environ..

[22] Kramer P.J. (1981). Carbon-dioxide concentration, photosynthesis, and dry-matter production. Bioscience.

[23] Bowes G. (1991). Growth at elevated CO_2_: Photosynthesis responses mediated through RuBisCO. Plant Cell Environ..

[24] Sage R.F., Monson R.K. (1999). C_4_ Plant Biology.

[25] Lara M.V., Andreo C.S., Shanker A. (2011). C_4_ plants adaptation to high levels of CO_2_ and to drought environments. Abiotic Stress in Plants-Mechanisms and Adaptations.

[26] Leakey A.D.B., Ainsworth E.A., Bernacchi C.J., Rogers A., Long S.P., Ort D.R. (2009). Elevated CO_2_ effects on plant carbon, nitrogen and water relations: six important lessons from FACE. J. Exp. Bot..

[27] Ghannoum O. (2009). C_4_ photosynthesis and water stress. Ann. Bot..

[28] Ripley B.S., Gilbert M.E., Ibrahim D.G., Osborne C.P. (2007). Drought constraints on C_4_ photosynthesis: Stomatal and metabolic limitations in C_3_ and C_4_ subspecies of *Alloteropsis semialata*. J. Exp. Bot..

[29] Chaves M.M., Maroco J.P., Pereira J.S. (2003). Understanding plant responses to drought-from genes to the whole plant. Funct. Plant Biol..

[30] Ribas-Carbo M., Taylor N.L., Giles L., Busquets S., Finnegan P.M., Day D.A., Lambers H., Medrano H., Berry J.A., Flexas J. (2005). Effects of water stress on respiration in soybean leaves. Plant Physiol..

[31] Ort D.R., Baker N.R. (2002). A photoprotective for O_2_ as an alternative electron sink in photosynthesis. Curr. Opin. Plant Biol..

[32] Sanda S., Yoshida K., Kuwano M., Kawamura T., Munekage Y.N., Akashi K., Yokota A. (2011). Responses of the photosynthetic electron transport system to excess light energy caused by water deficit in wild watermelon. Physiol. Plant..

[33] Flexas J., Medrano H. (2002). Drought-inhibition of photosynthesis in C_3_ plants: Stomatal and non-stomatal limitations revisited. Ann. Bot..

[34] Wang H., Zhang H., Li Z. (2007). Analysis of gene expression profile induced by water stress in upland rice (*Oryza sativa* L. var. IRAT109) seedlings using subtractive expressed sequence tags library. J. Integr. Plant Biol..

[35] Rabello A.R., Guimarães C.M., Rangel P.H.N., Silva F.R., Seixas D., Souza E., Brasileiro A.C.M., Spehar C.R., Ferreira M.E., Mehta A. (2008). Identification of drought-responsive genes in roots of upland rice (*Oryza sativa* L.). BMC Genom..

[36] Moumeni A., Satoh K., Kondoh H., Asano T., Hosaka A., Venuprasad R., Serraj R., Kumar A., Leung H., Kikuchi S. (2011). Comparative analysis of root transcriptome profiles of two pairs of drought-tolerant and susceptible rice near-isogenic lines under different drought stress. BMC Plant Biol..

[37] Wang D., Pan Y., Zhao X., Zhu L., Fu B., Li Z. (2011). Genome-wide temporal-spatial gene expression profiling of drought responsiveness in rice. BMC Genom..

[38] Hazen S.P., Pathan M.S., Sanchez A., Baxter I., Dunn M., Estes B., Chang H.S., Zhu T., Kreps J.A., Nguyen H.T. (2005). Expression profiling of rice segregating for drought tolerance QTLs using a rice genome array. Funct. Integr. Genom..

[39] Bray E.A. (2004). Genes commonly regulated by water-deficit stress in *Arabidopsis thaliana*. J. Exp. Bot..

[40] Dinneny J.R., Long T.A., Wang J.Y., Jung J.W., Mace D., Pointer S., Barron C., Brady S.M., Schiefelbein J., Benfey P.N. (2008). Cell identity mediates the response of Arabidopsis roots to abiotic stress. Science.

[41] Degenkolbe T., Do P.T., Zuther E., Repsilber D., Walther D., Hincha D.K., Köhl K.I. (2009). Expression profiling of rice cultivars differing in their tolerance to long-term drought stress. Plant Mol. Biol..

[42] Gorantla M., Babu P.R., Lachagari V.B.R., Reddy A.M.M., Wusirika R., Jeffrey L., Bennetzen J.L., Reddy A.R. (2007). Identification of stress-responsive genes in an indica rice (*Oryza sativa* L.) using ESTs generated from drought-stressed seedlings. J. Exp. Bot..

[43] Reddy A.R., Ramakrishna W., Sekhar A.C., Ithal N., Babu P.R., Bonaldo M.F., Soares M.B., Bennetzen J.L. (2002). Novel genes are enriched in normalized cDNA libraries from drought-stressed seedlings of rice (*Oryza sativa* L. subsp. indica cv. Nagina 22). Genome.

[44] Yang L., Zheng B., Mao C., Qi X., Liu F., Wu P. (2004). Analysis of transcripts that are differentially expressed in three sectors of the rice root system under water deficit. Mol. Gen. Genom..

[45] Zhou J., Wang X., Jiao Y., Qin Y., Liu X., He K., Chen C., Ma L., Wang J., Xiong L. (2007). Global genome expression analysis of rice in response to drought and high-salinity stresses in shoot, flag leaf, and panicle. Plant Mol. Biol..

[46] Ji K., Wanga Y., Sunb W., Louc Q., Meic H., Shena S., Chen H. (2012). Drought-responsive mechanisms in rice genotypes with contrasting drought tolerance during reproductive stage. J. Plant Physiol..

[47] Zhang C., Zhang L., Zhang S., Zhu S., Wu P., Chen Y., Li M., Jiang H., Wu G. (2015). Global analysis of gene expression profiles in physic nut (*Jatropha curcas* L.) seedlings exposed to drought stress. BMC Plant Biol..

[48] Hayano-Kanashiro C., Calderon-Vazquez C., Ibarra-Laclette E., Herrera-Estrella L., Simpson J. (2009). Analysis of gene expression and physiological responses in three Mexican maize landraces under drought stress and recovery irrigation. PLoS ONE.

[49] Osakabe Y., Osakabe K., Shinozaki S., Tran L.-S.P. (2014). Response of plants to water stress. Front. Plant Sci..

[50] Ramachandra Reddy A., Chaitanva K.V., Vivekanandan M. (2004). Drought-induced responses of photosynthesis and antioxidant metabolism in higher palnts. J. Plant Physiol..

[51] Ali G.H., Komatsu S. (2006). Proteomic analysis of rice leaf sheath during drought stress. J. Proteome Res..

[52] Deeba F., Pandey A.K., Ranjan S., Mishra A., Singh R., Sharma Y.K., Shirke P.A., Pandey V. (2012). Physiological and proteomic responses of cotton (*Gossypium herbaceum* L.) to drought stress. Plant Physiol. Biochem..

[53] Kosmala A., Perlikowski D., Pawłowicz I., Rapacz M. (2012). Changes in the chloroplast proteome following water deficit and subsequent watering in a high- and a low-drought-tolerant genotype of *Festuca arundinacea*. J. Exp. Bot..

[54] Kamal A.H., Cho K., Choi J.-S., Jin Y., Park C.-S., Lee J.S., Woo S.H. (2013). Patterns of protein expression in water-stressed wheat chloroplasts. Biol. Plant..

[55] Blödner C., Majcherczyk A., Kües U., Polle A. (2007). Early drought-induced changes to the needle proteome of Norway spruce. Tree Physiol..

[56] Praxedes S.C., DaMatta F.M., Loureiro M.E., Ferrão M.A.G., Cordeiro A.T. (2006). Effects of long-term soil drought on photosynthesis and carbohydrate metabolism in mature robusta coffee (*Coffea canephora* Pierre var. *kouillou*) leaves. Environ. Exp. Bot..

[57] Faghani E., Gharechahi J., Komatsu S., Mirzaei M., Khavarinejad R.A., Najafi F., Farsad L.K., Salekdeh G.H. (2015). Comparative physiology and proteomic analysis of two wheat genotypes contrasting in drought tolerance. J. Proteom..

[58] Zörb C., Herbst R., Forreiter C., Schubert S. (2009). Short-term effects of salt exposure on the maize chloroplast protein pattern. Proteomics.

[59] Ferreira S., Hjernø K., Larsen M., Wingsle G., Larsen P., Fey S., Roepstorff P., Salomé Pais M. (2006). Proteome profiling of *Populus euphratica* Oliv. upon heat stress. Ann. Bot..

[60] Han F., Chen H., Li X.J., Yang M.F., Liu G.S., Shen S.H. (2009). A comparative proteomic analysis of rice seedlings under various high-temperature stresses. Biochim. Biophys. Acta.

[61] Bertolde F.Z., Almeida A.A., Pirovani C.P. (2014). Analysis of gene expression and proteomic profiles of clonal genotypes from *Theobroma cacao* subjected to soil flooding. PLoS ONE.

[62] Gao F., Zhou Y., Zhu W., Li X., Fan L., Zhang G. (2009). Proteomic analysis of cold stress-responsive proteins in *Thellungiella rosette* leaves. Planta.

[63] Alam I., Sharmin S.A., Kim K.H., Yang J.K., Choi M.S., Lee B.H. (2010). Proteome analysis of soybean roots subjected to short-term drought stress. Plant Soil.

[64] Munns R. (1993). Physiological processes limiting plant growth in saline soils: Some dogmas and hypotheses. Plant Cell Environ..

[65] Chen X., Wang Y., Li J., Jiang A., Cheng Y., Zhang W. (2009). Mitochondrial proteome during salt stress-induced programmed cell death in rice. Plant Physiol. Biochem..

[66] Sobhanian H., Razavizadeh R., Nanjo Y., Ehsanpour A.A., RastgarJazii F., Motamed N., Komatsu S. (2010). Proteome analysis of soybean leaves, hypocotyls and roots under salt stress. Proteome Sci..

[67] Sobhanian H., Aghaei K., Komatsu S. (2011). Changes in the plant proteome resulting from salt stress: Toward the creation of salt-tolerant crops?. J. Proteom..

[68] Kim D.W., Rakwal R., Agrawal G.K., Jung Y.H., Shibato J., Jwa N.S. (2005). A hydroponic rice seedling culture model system for investigating proteome of salt stress in rice leaf. Electrophoresis.

[69] Arbona V., Manzi M., Ollas C., Gómez-Cadenas A. (2013). Metabolomics as a tool to investigate abiotic stress tolerance in plants. Int. J. Mol. Sci..

[70] Lei Y., Zheng Y., Dai K., Duan B., Cai Z. (2014). Different responses of photosystem I and photosystem II in three tropical oilseed crops exposed to chilling stress and subsequent recovery. Trees.

[71] Huang W., Zhang S.B., Cao K.F. (2010). Stimulation of cyclic electron flow during recovery after chilling-induced photoinhibition of PSII. Plant Cell Physiol..

[72] Sharma P., Sharma N., Deswal R. (2005). The molecular biology of the low temperature response in plants. Bioessays.

[73] Sage R.F., McKown A.D. (2006). Is C_4_ photosynthesis less phenotypically plastic than C_3_ photosynthesis?. J. Exp. Bot..

[74] Kimura M., Yamamoto Y.Y., Seki M., Sakurai T., Sato M., Abe T., Yoshida S., Manabe K., Shinozaki K., Matsui M. (2003). Identification of Arabidopsis genes regulated by high light-stress using cDNA microarray. Photochem. Photobiol..

[75] Murchie E.H., Hubbart S., Peng S., Horton P. (2005). Acclimation of photosynthesis to high irradiance in rice: Gene expression and interactions with leaf development. J. Exp. Bot..

[76] Zhang S., Scheller H.V. (2004). Photoinhibition of photosystem I at chilling temperature and subsequent recovery in *Arabidopsis thaliana*. Plant Cell Physiol..

[77] Barth C., Krause G.H., Winter K. (2001). Responses of photosystem I compared with photosystem II to high-light stress in tropical shade and sun leaves. Plant Cell Environ..

[78] Wang X., Peng Y., Singer J.W., Fessehaie A., Krebs S.L., Rajeev Arora R. (2009). Seasonal changes in photosynthesis, antioxidant systems and ELIP expression in a thermonastic and non-thermonastic Rhododendron species: A comparison of photoprotective strategies in overwintering plants. Plant Sci..

[79] Uberegui E., Hall M., Lorenzo Ó., Schröder W.P., Balsera M. (2015). An Arabidopsis soluble chloroplast proteomic analysis reveals the participation of the Executer pathway in response to increased light conditions. J. Exp. Bot..

[80] Phee B.K., Cho J.H., Park S., Jung J.H., Lee Y.H., Jeon J.S., Bhoo S.H., Hahn T.R. (2004). Proteomic analysis of the response of Arabidopsis chloroplast proteins to high light stress. Proteomics.

[81] Voesenek L.A., Colmer T.D., Pierik R., Millenaar F.F., Peeters A.J. (2006). How plants cope with complete submergence. New Phytol..

[82] Hossain Z., López-Climent M.F., Arbona V., Pérez-Clemente R.M., Gómez-Cadenas A. (2009). Modulation of the antioxidant systemin citrus under waterlogging and subsequent drainage. J. Plant Physiol..

[83] Mutava R.N., Prince S.J., Syed N.H., Song L., Valliyodan B., Chen W., Nguyen H.T. (2015). Understanding abiotic stress tolerance mechanisms in soybean: A comparative evaluation of soybean response to drought and flooding stress. Plant Physiol. Biochem..

[84] Nanjo Y., Maruyama K., Yasue H., Yamaguchi-Shinozaki K., Shinozaki K., Komatsu S. (2011). Transcriptional responses to flooding stress in roots including hypocotyl of soybean seedlings. Plant Mol. Biol..

[85] Kamal A.H., Rashid H., Sakata K., Komatsu S. (2015). Gel-free quantitative proteomic approach to identify cotyledon proteins in soybean under flooding stress. J. Proteom..

[86] Ahsan N., Donnart T., Nouri M.Z., Komatsu S. (2010). Tissue-specific defense and thermo-adaptive mechanisms of soybean seedlings under heat stress revealed by proteomic approach. J. Proteome Res..

[87] Hasanuzzaman M., Nahar K., Alam M.M., Roychowdhury R., Fujita M. (2013). Physiological, biochemical, and molecular mechanisms of heat stress tolerance in plants. Int. J. Mol. Sci..

[88] Vani B., PardhaSaradhi P., Mohanty P. (2001). Alteration in chloroplast structure and thylakoid membrane composition due to *in vivo* heat treatment of rice seedlings: correlation with the functional changes. J. Plant Physiol..

[89] Morales D., Rodriguez P., Dellamico J., Nicolas E., Torrecillas A., Sanchez-Blanco M.J. (2003). High-temperature preconditioning and thermal shock imposition affects water relations, gas exchange and root hydraulic conductivity in tomato. Biol. Plant..

[90] Wang J.Z., Cui L.J., Wang Y., Li J.L. (2009). Growth, lipid peroxidation and photosynthesis in two tall fescue cultivars differing in heat tolerance. Biol. Plant..

[91] Song Y., Chen Q., Ci D., Shao X., Zhang D. (2014). Effects of high temperature on photosynthesis and related gene expression in poplar. BMC Plant Biol..

[92] Hancock R.D., Morris W.L., Ducreux L.J., Morris J.A., Usman M., Verrall S.R., Fuller J., Simpson C.G., Zhang R., Hedley P.E. (2014). Physiological, biochemical and molecular responses of the potato (*Solanum tuberosum* L.) plant to moderately elevated temperature. Plant Cell Environ..

[93] Kangasjärvi S., Nurmi M., Tikkanen M., Aro E.M. (2009). Cell-specific mechanisms and systemic signalling as emerging themes in light acclimation of C_3_ plants. Plant Cell Environ..

[94] Hase T., Schürmann P., Knaff D.B., Golbeck J.H. (2006). The Light-Driven Plastocyanin: Ferredoxin Oxidoreductase. Photosystem I.

[95] Fey V., Wagner R., Bräutigam K., Pfannschmidt T. (2005). Photosynthetic redox control of nuclear gene expression. J. Exp. Bot..

[96] Abdallah F., Salamini F., Leister D. (2000). A prediction of the size and evolutionary origin of the proteome of chloroplasts of Arabidopsis. Trends Plant Sci..

[97] Singh R., Singh S., Parihar P., Singh V.P., Prasad S.M. (2015). Retrograde signaling between plastid and nucleus: A review. J. Plant Physiol..

[98] Giró M., Ceccoli R.D., Poli H.O., Carrillo N., Lodeyro A.F. (2011). An *in vivo* system involving co-expression of cyanobacterial flavodoxin and ferredoxin-NADP^+^reductase confers increased tolerance to oxidative stress in plants. FEBS Open Biol..

[99] Mao X., Jia D., Li A., Zhang H., Tian S., Zhang X., Jia J., Jing R. (2011). Transgenic expression of TaMYB2A confers enhanced tolerance to multiple abiotic stresses in Arabidopsis. Funct. Integr. Genom..

[100] Su J., Hirji R., Zhang L., He C., Selvaraj G., Wu R. (2006). Evaluation of the stress-inducible production of choline oxidase in transgenic rice as a strategy for producing the stress-protectant glycine betaine. J. Exp. Bot..

[101] Park E.J., Jeknić Z., Pino M.T., Murata N., Chen T.H. (2007). Glycinebetaine accumulation is more effective in chloroplasts than in the cytosol for protecting transgenic tomato plants against abiotic stress. Plant Cell Environ..

[102] Fan W., Zhang M., Zhang H., Zhang P. (2012). Improved tolerance to various abiotic stresses in transgenic sweet potato (*Ipomoea batatas*) expressing spinach betaine aldehyde dehydrogenase. PLoS ONE.

[103] Fu J., Momcilović I., Clemente T.E., Nersesian N., Trick H.N., Ristic Z. (2008). Heterologous expression of a plastid EF-Tu reduces protein thermal aggregation and enhances CO_2_ fixation in wheat (*Triticum aestivum*) following heat stress. Plant Mol. Biol..

[104] Wang L., Zhao C.M., Wang Y.J., Liu J. (2005). Overexpression of chloroplast-localized small molecular heat-shock protein enhances chilling tolerance in tomato plant. J. Plant Physiol. Mol. Biol..

[105] Zhang H., Mao X., Wang C., Jing R. (2010). Overexpression of a common wheat gene TaSnRK2.8 enhances tolerance to drought, salt and low temperature in Arabidopsis. PLoS ONE.

[106] Brini F., Hanin M., Mezghani I., Berkowitz G.A., Masmoudi K. (2007). Overexpression of wheat Na^+^/H^+^antiporter TNHX1 and H^+^-pyrophosphatase TVP1 improve salt- and drought-stress tolerance in *Arabidopsis thaliana* plants. J. Exp. Bot..

[107] He C., Yan J., Shen G., Fu L., Holaday A.S., Auld D., Blumwald E., Zhang H. (2005). Expression of an Arabidopsis vacuolar sodium/proton antiporter gene in cotton improves photosynthetic performance under salt conditions and increases fiber yield in the field. Plant Cell Physiol..

[108] Aharon R., Shahak Y., Wininger S., Bendov R., Kapulnik Y., Galili G. (2003). Overexpression of a plasma membrane aquaporin in transgenic tobacco improves plant vigor under favorable growth conditions but not under drought or salt stress. Plant Cell.

[109] Kono M., Terashima I. (2014). Long-term and short-term responses of the photosynthetic electron transport to fluctuating light. J. Photochem. Photobiol. B.

[110] Muneer S., Park Y.G., Manivannan A., Soundararajan P., Jeong B.R. (2014). Physiological and proteomic analysis in chloroplasts of *Solanum lycopersicum* L. under silicon efficiency and salinity stress. Int. J. Mol. Sci..

[111] Turkina M.V., Kargul J., Blanco-Rivero A., Villarejo A., Barber J., Vener A.V. (2006). Environmentally modulated phosphoproteome of photosynthetic membranes in the green alga *Chlamydomonas reinhardtii*. Mol. Cell. Proteom..

[112] Baena-Gonzalez E., Rolland F., Thevelein J.M., Sheen J. (2007). A central integrator of transcription networks in plant stress and energy signalling. Nature.

[113] Huber S.C. (2007). Exploring the role of protein phosphorylation in plants: from signalling to metabolism. Biochem. Soc. Trans..

[114] Liu X.D., Shen Y.G. (2004). NaCl-Induced phosphorylation of light harvesting chlorophyll a/b proteins in thylakoid membranes from the halotolerant green alga, *Dunaliella salina*. FEBS Lett..

[115] Aro E.M., Suorsa M., Rokka A., Allahverdiyeva Y., Paakkarinen V., Saleem A., Battchikova N., Rintamäki E. (2005). Dynamics of photosystem II: A proteomic approach to thylakoid protein complexes. J. Exp. Bot..

